# Relationship between Auditory Evoked Potentials and Circadian Preference in Patients with Major Depressive Episodes

**DOI:** 10.3390/brainsci10060370

**Published:** 2020-06-12

**Authors:** Young-Min Park

**Affiliations:** Department of Psychiatry, Ilsan Paik Hospital, Inje University College of Medicine, Goyang 10380, Korea; i0205@paik.ac.kr

**Keywords:** major depressive disorder, childhood trauma, circadian preference, morningness, eveningness, circadian rhythm

## Abstract

Mood disorders often accompany circadian rhythm abnormalities. The serotonergic system (STS) is related to mood and circadian rhythm. This study aimed to test whether serotonergic neurotransmission, using the loudness dependence of auditory evoked potential (LDAEP), is associated with circadian preference in patients with major depressive disorder (MDD). Depression severity was assessed in 18–65-year-old outpatients (*n* = 48) using the Beck Depression Inventory scores and Hamilton Depression Rating Scale at baseline. Additionally, various scales, including the Korean version of the Composite Scale of Morningness (K-CSM), Korean version of the Mood Disorder Questionnaire (K-MDQ), and Korean version of the Childhood Trauma Questionnaire (K-CTQ), were used. LDAEP was also measured at baseline. The subjects were divided into three groups according to the circadian preference using total K-CSM scores (morningness (*n* = 10) vs intermediate (*n* = 19) vs. eveningness (*n* = 19)) and two groups according to median based on each K-CSM score, respectively (higher K-CSM (*n* = 25) vs. lower K-CSM (*n* = 23)). The bipolarity, suicidality, and age at onset differed among the three groups. Impulsivity, depression severity, suicidality, hopelessness, bipolarity, frequency of emotional abuse, and age at onset differed between the two group divisions. Thus, the STS might serve as the mediator between the circadian system and mood.

## 1. Introduction

Mood disorders often accompany abnormalities in circadian rhythms [1,2]. Those abnormalities include advanced or delayed circadian rhythm [2]. Some investigators found that patients with depression exhibit differences in circadian preference from the normal population [3]. Depression has especially been related to evening preference [4]. Additionally, a recent study found that the group with evening preference had a higher severity of childhood trauma, depression, and bipolarity than the group with morning preference [5,6]. Another study found that evening preference is more prominent in patients with bipolar disorder (BD) than in those with major depressive disorders (MDD), suggesting that circadian rhythm disturbance is a biological marker for BD [2].

There were some attempts to demonstrate these circadian preferences using genetics. Several polymorphisms in CLOCK or PER3 genes have been investigated as possible genetic factors associated with the endophenotype of circadian rhythm disturbance in healthy individuals [7]. In addition to CLOCK genes, the serotonergic system (STS) was directly related to the circadian rhythm [8]. A recent animal study has found that the STS promotes sleep, and STS disruption reduces both sleep and the homeostatic response to sleep deprivation [9]. This study countered the hypothesis that the STS plays a major role in awakening rather than sleep. Furthermore, some investigators found a significant association between 5-HTTLPR polymorphism and circadian preference [10]. Thus, it seems that the STS could affect circadian rhythm and preference. However, there has been no study investigating the relationship between circadian preference and central serotonergic activity using event-related potentials.

The loudness dependence of auditory evoked potential (LDAEP) is an event-related potential by auditory stimuli and is calculated using the N100 and P200 amplitudes [11,12]. LDAEP is inversely correlated with central serotonergic activity [11,12]. Some animal and clinical studies have reported that the LDAEP is a biological marker of central serotonergic activity in mental disorder [13]. For example, a high pretreatment LDAEP is related to a better clinical response to antidepressant treatment in patients with MDD [14]. A high baseline LDAEP is also related to the suicidality of depressed patients [15]. In addition, LDAEP was found to be linked to the duration of illness in patients with schizophrenia [16].

The aim of the current study was to determine if the serotonergic activity using LDAEP is associated with circadian preference in patients with MDD.

## 2. Materials and Methods

### 2.1. Subjects and Study Design

In total, 48 subjects aged 18–65 years with MDD according to DSM-IV–text revision criteria, being a part of the Korean MDD cohort for suicide prevention (KOMDD study) [17] with the data of LDAEP and circadian preference, were selected. The inclusion criteria for the KOMDD study were (1) total Hamilton Depression Rating Scale (HAMD) and Beck Depression Inventory (BDI) scores exceeding 17 or 19, respectively, at pretreatment, and (2) no history of antidepressant administration during the 12 weeks before their first visit to our hospital. The exclusion criteria were (1) a previous hypomanic episode, (2) high suicide risk, (3) history of brain trauma or organic brain disease or (4) neurological disease. These exclusion criteria were applied to investigate the response of the MDD cohort to antidepressants in the KOMDD study. Depression severity was assessed using the Hamilton Depression Rating Scale (HAMD) [18] and Beck Depression Inventory (BDI) [19] scores at baseline. Additionally, various scales including the Hamilton Anxiety Rating Scale (HAMA) [20], Barratt Impulsiveness Scale (BIS) [21], Korean version of the Mood Disorder Questionnaire (K-MDQ) [22], Korean version of the Composite Scale of Morningness (K-CSM) [23], Korean version of the Childhood Trauma Questionnaire (K-CTQ) [24], Beck Scale for Suicide Ideation (BSS) [25], and Beck Hopelessness Scale (BHS) [26] were also used. Furthermore, the LDAEP was measured at baseline. The subjects were divided into morningness, intermediate, and eveningness groups according to the circadian preference based on the K-CSM. Additionally, the subjects were divided into lower and higher K-CSM groups according to the median based on the K-CSM.

The study protocol was approved by the ethics committee of Ilsan Paik Hospital (ethic code: IB-3-1105-014, in June 2011), and written informed consent to participate was obtained from all patients before commencing the investigations. All investigations were performed in accordance with the Declaration of Helsinki 1975, revised in 2013.

### 2.2. Scales

The K-CSM was derived from the widely used Horne–Ostberg scale [27]. Several studies have demonstrated good test–retest reliability and adequate external validity for the K-CSM [23,28,29]. The K-CTQ is a self-report questionnaire with five subscales of childhood abuse or neglect experience: physical abuse (PA), emotional abuse (EA), sexual abuse (SA), physical neglect (PN), and emotional neglect (EN). Each subscale consists of five items that are rated on a 5-point scale, from 1 (never true) to 5 (very often true). Depression was evaluated using the BDI and K-MDQ from the medical records. The K-MDQ indicated subjects as positive for screening of bipolarity or emotional dysregulation (e.g., borderline personality) [30].

### 2.3. Electroencephalography Methods

The auditory processing consisted of 1000 stimuli with an interstimulus interval of 500–900 ms. Tones at 1000 Hz and with a duration of 80 ms (with 10 ms rise and fall times) were generated by the E-Prime software (Psychology Software Tools, Pittsburgh, PA, USA) at 5 intensities (55, 65, 75, 85, and 95 dB) via headphones (MDR-D777, Sony, Tokyo, Japan). Electroencephalography data were recorded from 64 scalp sites according to the international 10–20 system (impedance < 10 kohm) using an Auditory Neuroscan NuAmp amplifier (Compumedics USA, El Paso, TX, USA). Similar to our previous study [31], additional detailed methods were performed.

**Figure 1:**
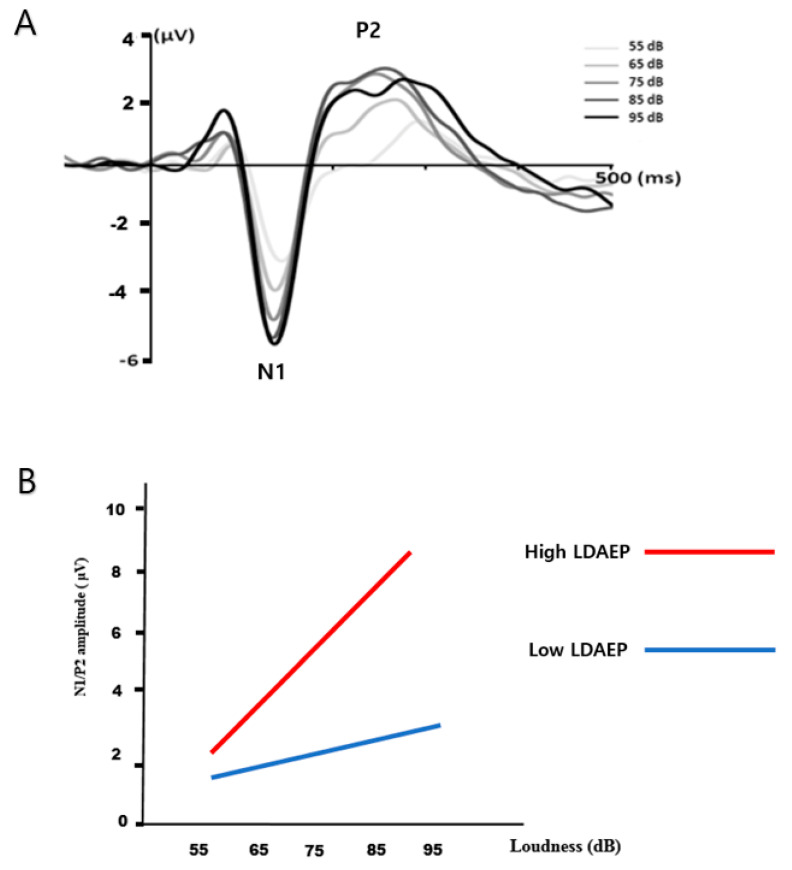
(**A**) Illustration of the loudness dependence of auditory evoked potential (LDAEP). The peak-to-peak N1/P2 amplitudes were measured for the five stimulus intensities, and the LDAEP was calculated as the slope of the linear-regression curve. (**B**) Illustration of a high and steep LDAEP (a large increase in N1/P2 amplitude with increasing loudness: a red line) and a low and shallow LDAEP (a small increase in N1/P2 amplitude with increasing loudness: a blue line).

The peak-to-peak N1/P2 amplitudes were measured for the five stimulus intensities, and the LDAEP was calculated as the slope of the linear regression curve (Figure 1).

### 2.4. Statistical Analysis

The subjects were divided into morningness, intermediate, and eveningness groups according to their circadian preference based on the K-CSM. In addition, the subjects were dichotomized according to the median K-CSM (the score of 31) into high and low K-CSM groups.

Clinical variables were identified to determine whether they are normally distributed by the Kolmogorov–Smirnov test. Analysis of variance (ANOVA), Kruskal–Wallis test, Student’s *t*-test, Mann–Whitney U-test, Chi-square test, and Pearson’s or Spearman’s correlation test were used to carry out group comparisons. Multivariate linear regression was used to identify the association between K-CTQ, K-MDQ, and K-CSM scores. All tests were two-tailed, and the cutoff for significant group differences was *p* < 0.05. The statistical analysis was carried out using the SALT 2.5 software packages (Istech Inc., Goyang, Korea).

## 3. Results

We observed 19 individuals in the evening type, 10 in the morning type, and 19 in the intermediate type. The K-CSM scores correlated positively with K-MDQ, BHS, and BSS scores, and negatively with age at onset (AAO). The subjects were divided into three groups according to the circadian preference (Table 1). The K-MDQ, BSS, BHS, and AAO scores differed among the three groups. Subjects were also divided into two groups according to the median value of K-CSM (Table 2). Groups with lower K-CSM scores had higher BIS, BDI, BSS, BHS scores, and earlier AAO than those with higher K-CSM scores.

Multivariate linear regression analyses were carried out to assess if low serotonergic activity affected the association with evening preference. High LDAEP and K-MDQ were independently associated with a low K-CSM score (Table 3). In other words, lower serotonergic activity was associated with toward-evening preference. Multivariate linear regression analyses were also performed to assess whether low serotonergic activity, childhood trauma, and evening preference affected the association with earlier age at onset. It revealed that LDAEP and K-CTQ, namely, lower central serotonergic activity and history of childhood abuse or neglect, were interactively associated with earlier age at onset (Table 4).

## 4. Discussion

The current study compared clinical variables between groups with different circadian preferences in patients with MDD. When the patients were divided into three groups according to their circadian preference, bipolarity, suicidality, and age at onset differed among the three groups. When the patients were divided into two groups according to the median value of K-CSM, impulsivity, depression severity, suicidality, hopelessness, resilience, frequency of emotional abuse, and age at onset differed between the two groups. In addition, the high LDAEP group and higher K-MDQ were independently associated with lower K-CSM score, indicating evening preference. LDAEP and K-CTQ, namely, central serotonergic strength and childhood abuse or neglect, were interactively associated with age at onset. Thus, our results revealed that depressed individuals with toward-evening preference had more severe psychopathology than those with toward-morning preference. Moreover, STS seems to serve as the mediator between childhood trauma, circadian disruption, and mood disorder.

Some studies suggest that trauma affects STS [32,33]. A meta-analysis revealed that the *s* allele of the 5-HTTLPR is related to a higher risk of developing MDD after experiencing childhood trauma [32]. The methylation of the 5-HT3 receptor was also associated with age at onset and clinical outcomes, including disease severity, the number of mood episodes, history of suicide attempts, and hospitalization in patients with BD [33]. The current study also revealed that lower central serotonergic activity and more severe childhood maltreatment were interactively associated with an earlier age at onset. Taken together, STS and childhood trauma might be closely related to each other. However, in a recent meta-analysis, some investigators did not find a positive interaction between stressful life events and the 5-HTTLPR genotype in the onset of MDD [34]. In addition, recent findings on the relationship between childhood trauma and polygenic risks in patients with MDD using genome wide association study are being reported [35,36]. Thus, the link between STS and childhood trauma is still inconclusive.

Studies have also been conducted to determine the association between STS and circadian rhythm. The loss of *Pet-1* that plays a critical role in 5-HT neuron development [37] disrupts the rhythms of locomotor activity and elongates the phase of suprachiasmatic nucleus (SCN) neuronal activity [8]. The circadian rhythmic release of cortisol leads to the synthesis of 5-HT [38,39], resulting in rhythmic 5-HT secretion within the SCN [40]. However, repetitive stressors attenuate the tryptophan hydroxylase 2 (TPH2) rhythm in the dorsal raphe nucleus for producing TPH [40,41], the rate-limiting enzyme in the production of 5-HT from L-tryptophan [42]. Finally, 5-HT depletion leads to the abnormalities of circadian rhythm [43]. Evidence indicating the relationship between STS and an evening preference has also been found. The evening types reported a worse treatment response to selective serotonin reuptake inhibitors than the morning types [44]. Administration of 5-HT agonist during the night induces phase advances in SCN neural activity [45,46]. Conversely, 5-HT at night inhibits the action of light on SCN [8]. Thus, 5-HT deficiency may disrupt or delay the circadian rhythms [47]. The current study also revealed that the higher LDAEP group was associated with lower K-CSM score (toward eveningness) following linear regression. Thus, 5-HT deficiency may play a role in delayed phase in mood disorders.

The research on LDAEPs has been widely investigated in the field of psychiatry and neurology for its relationships with the pathophysiology and psychopathology of neuropsychiatric disease and central serotonergic activity since Hegerl and Juckel discovered it [11]. Several studies have reported that the LDAEP was very useful as a central serotonergic marker [13,14,48,49]. Furthermore, some investigators recently found that LDAEP was significantly correlated with 5-HT_1A_ binding positively and with 5-HTT binding negatively in the temporal cortex using positron emission tomography [50]. Thus, LDAEP seems to be a potential central serotonergic biomarker, although large samples studies are needed.

**Figure 2:**
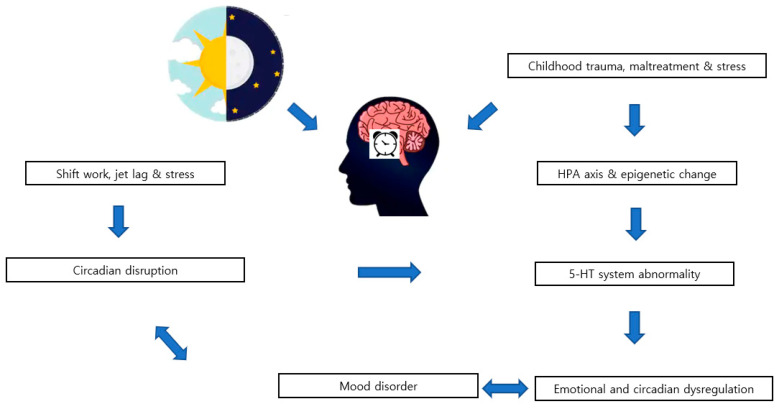
Schematic diagram indicating the relationship among the serotonergic system, circadian disruption, and mood disorder.

Taken together, it may be hypothesized that childhood trauma or maltreatment affects the HPA axis, which in turn affects the STS, causing emotional and circadian dysregulation, resulting in more severe psychopathology and circadian disruption (Figure 2). The STS may serve as the common link between stress, mood, and the circadian system [51].

This study has several limitations. Firstly, the sample size was small; therefore, studies involving larger samples are needed in the future. Secondly, circadian preference may differ from the authentic chronotype because in this study, it was investigated by only subjective scales, such as K-CSM. However, this is the first study to have investigated the relationship between serotonergic activity using LDAEP and circadian preference. Thirdly, detailed information on the STS could not be obtained from the LDAEP because it was measured through the electrodes from the scalp. However, these measurements are being compensated using several techniques, including standardized low- resolution brain electromagnetic tomography.

## 5. Conclusions

Low serotonergic activity was associated with evening preference. In addition, low serotonergic activity and childhood maltreatment were interactively associated with earlier onset. Thus, this study supports the hypothesis that the STS may serve as the common link between the circadian system and mood and that the interaction between STS and childhood maltreatment may contribute to pathophysiology of MDD.

## Figures and Tables

**Table 1:** Comparison of demographic and clinical variables among the morningness, intermediate, and eveningness groups classified according to circadian preference based on the Korean version of the Composite Scale of Morningness (K-CSM).

Variable	Morningness (*n* = 10)	Intermediate (*n* = 19)	Eveningness (*n* = 19)	*p*
Age at assessment, years	40.0 ± 13.9	41.1 ± 13.0	34.2 ± 12.9	0.24
^a^ Sex ratio, males/females	4/6	1/18	4/15	0.065
Age at onset, years	42.83 ± 7.99	32.90 ± 13.10	25.21 ± 11.45	< 0.01
^b^ Number of episodes	2.83 ± 4.02	5.05 ± 4.29	4.90 ± 4.47	0.11
^a^ Presence of bipolarity	8/2	16/3	5/14	< 0.001
LDAEP (µV/10 dB)	0.84 ± 0.53	1.38 ± 0.77	1.07 ± 0.73	0.14
BIS	84.67 ± 20.37	71.68 ± 21.37	84.42 ± 12.90	0.077
BDI	25.50 ± 10.19	26.58 ± 9.97	32.58 ± 6.56	0.057
^b^ BSS	8.67 ± 11.09	9.53 ± 7.84	17.90 ± 9.15	< 0.05
^b^ BHS	7.67 ± 6.35	8.68 ± 6.11	16.50 ± 3.13	< 0.001
HAMD	17.17 ± 7.08	18.79 ± 4.89	19.05 ± 5.79	0.77
HAMA	17.67 ± 7.34	20.11 ± 6.65	21.74 ± 6.79	0.43
^b^ K-MDQ	4.80 ± 1.75	4.47 ± 2.55	8.32 ± 3.13	< 0.001
K-CTQ	40.50 ± 14.52	50.63 ± 17.29	55.95 ± 22.80	0.13
^b^ Emotional abuse	6.60 ± 2.37	10.58 ± 5.00	12.26 ± 5.58	< 0.05
^b^ Physical abuse	7.70 ± 3.16	9.53 ± 4.82	11.26 ± 6.40	0.34
^b^ Sexual abuse	5.80 ± 1.14	5.79 ± 1.48	8.00 ± 5.13	0.71

Data are mean ± SD or percentage values. ^a^ Fisher’s exact test, ^b^ Kruskal–Wallis test. Abbreviations: K-CSM = Korean version of the Composite Scale of Morningness; LDAEP = loudness dependence of auditory evoked potential; BIS = Barratt Impulsiveness Scale; BDI = Beck Depression Inventory; BSS = Beck Scale for Suicide Ideation; BHS = Beck Hopelessness Scale; HAMD = Hamilton Depression Rating Scale; HAMA = Hamilton Anxiety Rating Scale; K-MDQ = Korean version of the Mood Disorder Questionnaire; K-CTQ = Korean version of the Childhood Trauma Questionnaire.

**Table 2:** Comparison of demographic and clinical variables between the two groups dichotomized according to the median K-CSM score.

Variable	Higher K-CSM (*n* = 25) (Toward Morningness)	Lower K-CSM (*n* = 23) (Toward Eveningness)	*p*
Age at assessment, years (mean ± SD)	40.5 ± 13.9	35.5 ± 12.3	0.20
^a^ Sex ratio, males/females	5/20	4/19	0.82
Age at onset, years	35.05 ± 13.42	27.17 ± 11.73	< 0.05
^b^ Number of episodes	4.24 ± 4.02	5.09 ± 4.59	0.52
^a^ Presence of bipolarity	22/3	7/16	< 0.001
LDAEP (µV/10 dB)	1.16 ± 0.74	1.12 ± 0.73	0.86
BIS	72.33 ± 21.33	85.00 ± 13.82	< 0.05
BDI	25.12 ± 9.67	32.65 ± 6.91	< 0.01
^b^ BSS	9.00 ± 8.49	17.90 ± 9.15	< 0.01
^b^ BHS	8.14 ± 6.05	15.32 ± 4.57	< 0.001
HAMD	19.41 ± 6.71	19.09 ± 5.69	0.62
HAMA	17.67 ± 7.34	21.70 ± 6.75	0.22
^b^ K-MDQ	4.40 ± 2.10	7.87 ± 3.24	< 0.001
K-CTQ	46.24 ± 16.17	55.39 ± 22.28	0.11
^b^ Emotional abuse	8.84 ± 4.43	12.13 ± 5.52	< 0.05
^b^ Physical abuse	9.04 ± 4.49	10.70 ± 6.09	0.43
^b^ Sexual abuse	5.92 ± 1.41	7.48 ± 4.79	0.93

Data are mean ± SD or percentage values. ^a^ Fisher’s exact test, ^b^ Mann–Whitney U-test. Abbreviations: K-CSM = Korean version of the Composite Scale of Morningness; LDAEP = loudness dependence of auditory evoked potential; BIS = Barratt Impulsiveness Scale; BDI = Beck Depression Inventory; BSS = Beck Scale for Suicide Ideation; BHS = Beck Hopelessness Scale; HAMD = Hamilton Depression Rating Scale; HAMA = Hamilton Anxiety Rating Scale; K-MDQ = Korean version of the Mood Disorder Questionnaire; K-CTQ = Korean version of the Childhood Trauma Questionnaire.

**Table 3:** Results of multiple linear regression analysis for the association between K-CSM scores and clinical variables.

Variables	Coefficient	SE	*t*	*p*
	K-CSM Score			
Age	0.023	0.13	0.17	0.87
Gender	−1.25	3.19	−0.39	0.70
BDI	−0.25	0.13	−1.91	0.064
K-MDQ	−1.71	0.38	−4.55	<0.001
K-CTQ	−0.01	0.063	−0.15	0.88
Age at onset	0.015	0.15	0.098	0.92
Groups based on low and high LDAEP	−7.56	2.51	−3.01	<0.01

Abbreviations: K-CSM = Korean version of the Composite Scale of Morningness; BDI = Beck Depression Inventory; K-MDQ = Korean version of the Mood Disorder Questionnaire; K-CTQ = Korean version of the Childhood Trauma Questionnaire; LDAEP = loudness dependence of auditory evoked potential.

**Table 4:** Results of multiple linear regression analysis for the association between age at onset and clinical variables.

Variables	Coefficient	SE	*t*	*p*
	Age at Onset			
Gender	0.097	5.38	0.018	0.99
K-CTQ	−0.26	0.20	−1.31	0.20
Types of circadian preference	−5.51	6.09	−0.90	0.37
LDAEP	−10.25	7.67	−1.34	0.19
Interaction of K-CTQ and LDAEP	−14.08	5.87	−2.40	<0.05

Abbreviations: K-CSM = Korean version of the Composite Scale of Morningness; K-CTQ = Korean version of the Childhood Trauma Questionnaire; LDAEP = loudness dependence of auditory evoked potential.
